# Linking enhancing and impairing effects of emotion—the case of PTSD

**DOI:** 10.3389/fnint.2013.00026

**Published:** 2013-05-20

**Authors:** Florin Dolcos

**Affiliations:** ^1^Psychology Department, University of Illinois at Urbana-ChampaignChampaign, IL, USA; ^2^Neuroscience Program, University of Illinois at Urbana-ChampaignChampaign, IL, USA; ^3^The Beckman Institute for Advanced Science and Technology, University of Illinois at Urbana-ChampaignChampaign, IL, USA

## Introduction

As illustrated by the present Research Topic, emotion that can either enhance or hinder various aspects of our cognition and behavior. For instance, the emotional charge of an event can increase attention to and memory for that event (Dolcos et al., 2012), whereas task-irrelevant emotional information may lead to increased distraction away from goal-relevant tasks (Iordan et al., 2013; see also Dolcos et al., 2011). Interestingly, sometimes these opposing effects of emotion co-occur. For example, hearing a gunshot may enhance memory for central aspects of what was happening at the time, while impairing memory for peripheral details (Christianson, 1992). It is also possible that increased distraction from ongoing goals produced by task-irrelevant emotional stimuli may lead to better memory for the distracting information itself. The co-occurrence of enhancing and impairing effects of emotion is probably most evident in affective disorders, where both of these opposing effects are exacerbated. Specifically, uncontrolled recollection of and rumination on distressing memories observed in depression and post-traumatic stress disorder (PTSD) may also lead to impaired cognition due to enhanced emotional distraction. Here, we illustrate an example based on evidence from studies of PTSD, pointing to the importance of investigating both enhancing and impairing effects of emotion, in elucidating the nature of alterations in the way emotion interacts with cognition in clinical conditions.

## Background: emotional and cognitive processing in PTSD

Changes in emotional and cognitive processing are critical features in PTSD patients, typically reflected in increased emotional reactivity and recollection of traumatic memories, along with impaired cognitive/executive control (Rauch et al., 2006; Shin and Liberzon, 2009; see also in this issue Brown and Morey, 2012; Hayes et al., 2012). Of particular note is emerging evidence concerning the neural correlates of alterations associated with the encoding of emotional memories (Hayes et al., 2011) and with the responses to task-irrelevant emotional distraction (Morey et al., 2009). These changes are reflected in regions associated with functions that may be enhanced (episodic memory) or impaired (working memory) by emotion—i.e., the medial temporal lobe (MTL) and dorsolateral prefrontal cortex (dlPFC), respectively. Here, we illustrate how understanding the changes associated with the way traumatic memories are formed and retrieved in PTSD (involving MTL areas) may clarify their impact on ongoing cognitive/executive processes (reflected in changes of dlPFC activity), when potential cues for traumatic memories are presented as task irrelevant distracters.

## The enhancing effect of emotion

Studies investigating the memory-enhancing effect of emotion in healthy participants point to the role of basic MTL mechanisms involving interactions between emotion-based regions (amygdala—AMY) and memory-related regions (hippocampus and associated parahippocampal cortices—HC, PHC) in the formation and retrieval of emotional memories (Dolcos et al., 2012). Neurobiological models of PTSD (Layton and Krikorian, 2002) propose that the development and maintenance of the disorder is linked to altered activity in the MTL during encoding of traumatic memories. Hence, intrusive recollection of traumatic memories observed in PTSD may be linked to dysfunction of the basic MTL mechanism identified in healthy participants as being responsible for the memory-enhancing effect of emotion (Dolcos et al., 2004). Specifically, processing of cues related to traumatic events may trigger recollection of traumatic memories, which due to dysfunctional interactions between AMY and the MTL memory system may engage a self-sustaining functional loop in which emotion processing in AMY may enhance recollection by increasing activity in HC; this, in turn, may intensify AMY activity as a result of re-experiencing the emotions associated with the recollected memories (Dolcos et al., 2005; McNally, 2006). On the other hand, there is also evidence suggesting a disconnect between the effects observed in AMY and their link to emotional or cognitive aspects of processing in PTSD patients. Specifically, while greater AMY activation is identified in studies of symptom provocation (Rauch et al., 2000; Hendler et al., 2003; Shin et al., 2004, 2005; Williams et al., 2006), such an effect is not observed in studies of cognitive processing (Shin et al., 2001; Clark et al., 2003; Bremner et al., 2004; Morey et al., 2008).

**Figure 1:**
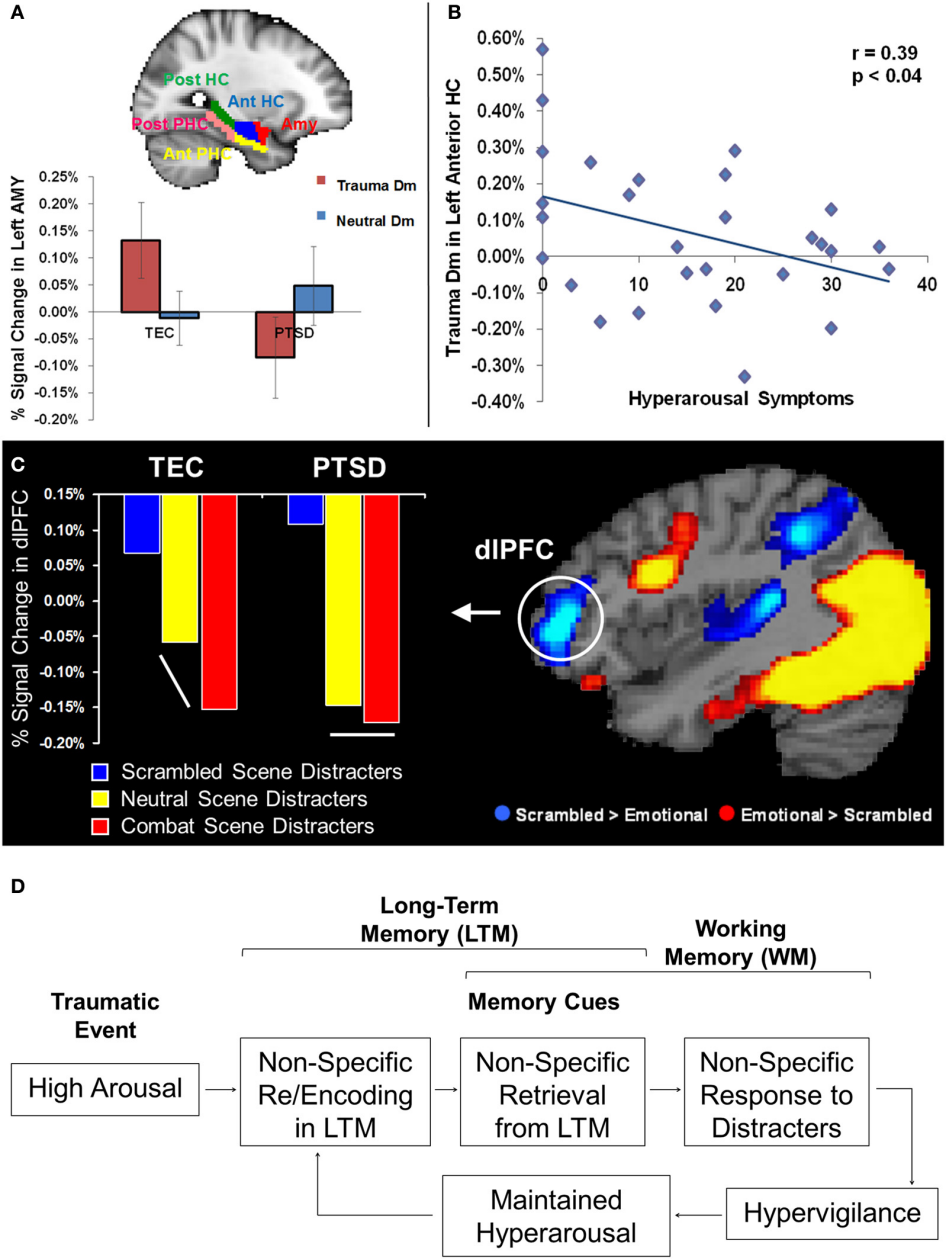
**(A–B)** Memory-Related Changes in the Medial Temporal Lobe Activity in PTSD. Reduced memory-related activity (Dm) in AMY for trauma-related pictures, in the PTSD group **(A)**; a similar effect was also observed in the HC (not shown). Reduced Dm for trauma-related pictures in the anterior HC linked to increased symptoms of arousal **(B)**. Dm, Difference due to Memory effect (brain activity for Remembered minus Forgotten items); PTSD, post-traumatic stress disorder; TEC, Trauma-Exposed Control group; AMY, Amygdala; HC, Hippocampus; PHC, Parahippocampal Cortex; Ant, Anterior; Post, Posterior. Error bars represent the standard error of means. Adapted from Hayes et al. (2011), with permission. **(C)** Evidence for Non-specific Response in dlPFC to Trauma-Related and Neutral Distracters in PTSD. Comparison of mean percentage signal change in dlPFC during the active maintenance period of a working memory task in the PTSD and Trauma-Exposed Control (TEC) groups point to a generalized dlPFC disruption of activation for salient task-irrelevant distracter scenes in the PTSD group, which showed an undifferentiated response in the dlPFC to combat and neutral distracters. The TEC group showed disruption in the same area, but specific to combat-related distraction. dlPFC, dorsolateral prefrontal cortex. Adapted from Morey et al. (2009), with permission. **(D)** Diagram illustrating a possible link between the impact of emotion on long-term memory and working memory in PTSD.

An important observation that has emerged in the PTSD literature may reconcile this apparent discrepancy. Specifically, there is evidence that memories for negative events in PTSD patients may be non-specific, gist-based, rather than detailed, context-based (McNally et al., 1994; Kaspi et al., 1995; Harvey et al., 1998). Gist refers to familiarity-based retrieval of memories for the general meaning of a situation or event, rather than recollection of specific contextual details (Tulving, 1985). Given that gist-based memories are often inaccurate (Roediger and McDermott, 1995; Wright and Loftus, 1998) and susceptible to enhanced rate of false alarms that may diminish or cancel an actual enhancing impact of emotion on memory (Dolcos et al., 2005), it may be the case that the basic AMY-MTL mechanisms typically responsible for the memory-enhancing effect of emotion are in fact attenuated in PTSD. Hence, this could explain the non-specific, gist-based, memories observed in these patients. This idea is supported by recent findings from a fMRI study using the subsequent memory paradigm with emotional stimuli in PTSD patients (Hayes et al., 2011), which showed reduced memory-related activity in the AMY-MTL system during memory encoding, and higher false alarm rates during retrieval, compared to a trauma exposed control (TEC) participants (Figure 1A). Moreover, the PTSD patients also lacked the anterior posterior dissociation along the longitudinal axis of the MTL, with respect to its involvement during successful encoding of emotional memories, which was initially identified in healthy participants (Dolcos et al., 2004), but such dissociation was preserved in the TEC group (Hayes et al., 2011). Together, these findings suggest a disorganization of the MTL mechanisms involved in the memory-enhancing effect of emotion in PTSD, which leads to inefficient encoding of information for trauma-related stimuli and subsequent non-specific gist-based retrieval.

## The impairing effect of emotion

Studies investigating the neural correlates of the impairing effect of task-irrelevant emotional distraction on cognitive performance identified distinct patterns of responses in emotion and cognitive control brain regions (i.e., increased activity in AMY and reduced activity in dlPFC, respectively), which are specific to emotional distraction (Dolcos et al., 2011). On the one hand, based on this evidence, increased emotional reactivity linked to changes in the AMY function in PTSD may lead to increased specific disruption of dlPFC activity by emotional distraction. On the other hand, there is evidence for a non-specific heightened sensitivity to both threatening and non-threatening stimuli in PTSD (Grillon and Morgan, 1999; Peri et al., 2000), which may explain increased distractibility to trauma related and unrelated stimuli alike.

The fact that information unrelated to the trauma may also be highly distracting in PTSD patients is consistent with the clinically observed symptom of *hypervigilance* in these patients (American Psychiatric Association, 2000), and with the evidence for non-specific encoding of trauma-related material discussed above (Hayes et al., 2011). Specifically, it is reasonable to expect that seemingly neutral stimuli that may remind of trauma could act as cues for non-specific retrieval of trauma-related information, which in turn may be as distracting as the trauma-related stimuli themselves. Evidence from a recent study of WM with trauma-related and non-related distraction is consistent with this idea (Morey et al., 2009). Using an adaptation of our WM task with emotional distraction (Dolcos and McCarthy, 2006), the study by Morey and colleagues investigated how trauma-related task-irrelevant emotional information modulates WM networks in PTSD. Similar to the study on memory encoding discussed above, recent post-9/11 war veterans were divided into a PTSD group and a TEC group. Functional MRI results showed that the PTSD group had greater trauma-specific activation than the control group in main emotion processing brain regions, including the AMY and ventrolateral PFC (vlPFC), as well as in brain regions susceptible to emotion modulation (e.g., fusiform gyrus—FG). However, the PTSD group also showed greater non-specific disruption of activity to combat-related and neutral task-irrelevant distracters in brain regions that subserve the ability to maintain focus on goal-relevant information, including the dlPFC. This suggests a more generalized dlPFC disruption in the PTSD group than in the control group, which showed disruption specific to the trauma-related distraction. The undifferentiated dlPFC response to combat and non-combat distracters in PTSD is consistent with the *hypervigilance* hypothesis that may explain enhanced response to and distracting effect of neutral stimuli (Figure 1C). This neural-level finding was complemented by the behavioral results, which showed lower overall working memory performance for task-irrelevant distracters scenes in the PTSD group, in the absence of a differential impact between combat-related and neutral distracters.

## The link between enhancing and impairing effects of emotion

Overall, the evidence from separate lines of investigations discussed above, regarding the neural changes in PTSD linked to dysfunctions in the recollection of traumatic events and the response to emotional distraction, converge toward the idea that non-specific response to emotional and neutral distraction may reflect retrieval distortions linked to inefficient initial encoding of trauma-related information. Namely, it is possible that the non-specific disruption of the dlPFC activity by trauma-related and neutral distraction is linked to the retrieval of the traumatic memories triggered by non-specific cues, which may also contribute to the perpetuation of the state of *hyperarousal* observed in these patients (Figure 1D). Moreover, it is also possible that the source of these effects may be linked to elevated arousal during the initial exposure to traumatic events. Consistent with this idea, in addition to showing non-specific activity to subsequently remembered items in AMY and MTL memory system in PTSD, the study by Hayes and colleagues discussed above (Hayes et al., 2011) also identified a negative co-variation of memory-related hippocampal activity for trauma-related items with scores of hyperarousal symptoms, as measured with the Clinician-Administered PTSD Scale (Figure 1B). In other words, participants who had greater hyperarousal scores also had reduced memory-related activity during the encoding of trauma-related pictures. This finding is consistent with evidence for an inverted U-shaped function in the hippocampus as a function of stress (Nadel and Jacobs, 1998) and provides a possible explanation for the non-specific effects observed in the tasks assessing emotional memory for trauma-related cues and their undifferentiated impact on goal-relevant processing when presented as task-irrelevant distraction. Consistent with the role of the initial arousal in these effects, PTSD patients also showed relatively greater activity for forgotten items, which may be linked to AMY hyperactivity leading to later forgetting of those items (Hayes et al., 2011).

## Conclusion

In summary, available evidence from investigations of PTSD patients points to general and specific emotional and cognitive disturbances that are linked to alterations in the neural circuitry underlying emotion-cognition interactions. This evidence suggests that reduction of AMY and HC signals for trauma-related cues may underlie non-specific encoding of gist-based representations instead of specific and detailed contextual details of the trauma-related memories. This, in turn, may be linked to symptoms of *hypervigilance* and non-specific responses to trauma-related distraction, which contributes to the maintenance of a *hyperarousal* state (Figure 1D). This evidence also highlights the importance of investigating both the enhancing and the impairing effects of emotion, in understanding the changes associated with affective disorders, where both effects are intensified. Collectively, these findings point to the importance of investigating both of these opposing effects of emotion within the same clinical group, to complement similar approaches in healthy participant concomitantly investigating the enhancing and impairing effects of emotion on cognitive processes (Shafer and Dolcos, 2012; Dolcos et al., 2013).
